# Nursing education in conflict: How intersectionality impacts access to educational opportunities?

**DOI:** 10.3389/fmed.2026.1804741

**Published:** 2026-05-11

**Authors:** Laura Hinsche, Martina Hasseler, Tim Tischendorf, Tom Schaal

**Affiliations:** 1 Faculty of Healthcare, Ostfalia University of Applied Sciences, Wolfsburg, Germany; 2 Faculty of Health and Healthcare Sciences, University of Applied Sciences Zwickau, Zwickau, Germany

**Keywords:** inequalities, intersectional frameworks, intersectionality, nursing education, scoping review

## Abstract

**Background:**

Nursing, as a pillar of healthcare, must train a diversifying workforce under increasingly complex conditions. Access to education is affected by intersectional factors including gender, international biographie, socio-economic status, and hierarchical structures, yet systematic analyses remain limited.

**Methods:**

A PRISMA-based scoping review was conducted using PubMed, Ebsco CINAHL, PeDocs, CareLit, Livivo, and Google Scholar. Seventy studies were screened, with eight articles thoroughly analyzed for conceptual insights.

**Results:**

Intersectional barriers substantially hinder access to educational and qualification opportunities in nursing, disproportionately affecting marginalized groups. Hierarchical structures, Eurocentric curricula, and inadequately adapted digital strategies exacerbate these disparities and perpetuate discriminatory practices that negatively impact both carers and patients.

**Conclusion:**

Traditional deficit-oriented approaches focus on perceived adaptation issues among marginalized groups rather than on the structural barriers within the healthcare system. Early integration of intersectional perspectives in nursing education is essential for fostering ethical awareness, challenging power imbalances, and empowering professionals to dismantle discriminatory practices. Comprehensive curricular reforms that address racism, gender dynamics, and socio-economic inequities can promote inclusivity, improve professional retention, and enhance patient care outcomes. Systematically integrating intersectional frameworks into nursing education is crucial for reducing systemic inequities and catalyzing long-term structural change in the healthcare system.

## Background

Nursing, as a central component of the healthcare system, faces the challenge of qualifying a diverse and expanding professional group under increasingly complex conditions. However, access to educational programs in the nursing sector is influenced by intersectional factors such as gender, international background, socio-economic status and existing hierarchical structures (1). Marginalized groups in particular face structural barriers that limit their professional development (2).

Intersectionality has been defined as “the interaction between gender, race, and other categories of difference in individual lives, social practices, institutional arrangements, and cultural ideologies, and the outcomes of these interactions in terms of power” (3). The concept gained prominence through the work of the African-American legal scholar Kimberlé Crenshaw, who coined the term “intersectionality” to describe the overlapping and mutually reinforcing forms of discrimination experienced by Black women at the intersection of racism and sexism (4). Rather than treating categories such as gender, ethnicity, class or migration as isolated factors, an intersectional perspective examines how these dimensions interact and are embedded in broader relations of power and domination.

Subsequent scholarship has emphasized that even emancipatory movements may reproduce exclusion when they primarily reflect the perspectives of socially privileged groups: bell hooks, for example, argued that feminist movements often center the experiences of white women while marginalizing black women and reproducing unequal divisions of labor (5). Similarly, Patricia Hill Collins described these dynamics as an “interlocking system of oppression” shaped by race, class, gender, sexuality and nation. She argued that dominant groups frequently present their own partial perspectives as universal truths (6). In this sense, intersectionality is a critical analytical framework that reveals structural inequalities and examines how institutional arrangements perpetuate exclusion (7, 8). This perspective is particularly relevant in nursing and healthcare, where educational and professional opportunities are shaped by various interrelated factors, such as gender, age, socioeconomic status, and international background (9). Around one quarter of nursing professionals in Germany have an international background, and approximately 80% of this group are women (10). This group frequently encounters overlapping forms of disadvantage, including delayed recognition of qualifications, language-related exclusion, gendered care responsibilities, and limited access to further education and career development. An intersectional perspective therefore provides an important framework for understanding how inequalities are reproduced within nursing education and healthcare institutions.

Moreover, intersectionality is increasingly relevant in the context of digitalization and artificial intelligence. This is because language-based systems, digital technologies and AI applications may reproduce existing patterns of exclusion if they are developed primarily from dominant perspectives and without the inclusion of marginalized voices. Nursing professionals experiencing multiple forms of marginalization – for instance, due to gender, international background, or socio-economic disadvantage – may therefore be particularly vulnerable to algorithmic exclusion (11).

At the same time, the International Council of Nurses (ICN) and the World Health Organization (WHO) have repeatedly emphasized that most health systems worldwide are increasingly dependent on the international recruitment of nurses and nursing students to cover staffing needs (12). Against this backdrop, the intersectional challenges faced by internationally mobile nurses are of high relevance not only in Germany but in almost all regions of the world, where nursing shortages represent a critical risk to the stability and equity of healthcare provision.

In addition to the “classic areas of tension” in the dimensions of professionalism, ethical conduct, organization of everyday working life and knowledge of national professional standards (13), nursing staff from marginalized groups often experience various forms of discrimination and devaluation, both from patients and from their own professional group (14, 15). These experiences of discrimination begin in the context of nursing qualifications and continue institutionally through teaching. Due to a lack of reflection and engagement with the issue, all actors in the structures continue to reproduce it (15). In addition to the established areas of tension in nursing – such as professionalism, ethical conduct, organizational structures, and knowledge of national standards (13) – the findings reveal further structural and educational conflicts. These include tensions between workforce expansion and quality of care, as well as the well-documented theory – practice gap, which often leads to moral stress (16, 17) and disillusionment among trainees (18). Furthermore, trainees experience role conflicts between their position as learners and their function as part of the workforce, alongside challenges in balancing training with private care responsibilities, particularly in the context of the gender care gap. Additional tensions arise from discrimination and language-related barriers within educational settings (14), as well as from hierarchical power structures that shape access to support and participation. Finally, the increasing digitalization of nursing introduces a further tension between technological innovation and the relational core of care practice (19).

Although these factors are known, there is a lack of systematic analysis of how intersectionality specifically impacts access to education and qualification opportunities in nursing. The aim of this critical review is to systematically record and analyze the existing literature to answer the following research question: *How does intersectionality in nursing impact access to education and educational opportunities?* The critical review provides a comprehensive overview of current findings and existing research gaps.

## Methods

The literature search was based on the PRISMA scoping review process. Several databases and websites were systematically searched, including PubMed via MEDLINE, Ebsco CINAHL, PeDocs, CareLit and Livivo. A supplementary search was conducted using Google Scholar. Specific search terms were defined as part of the topic selection process and initial research, including “intersectional,” “intersectionality” and “nursing education”. The search strategies were adapted for each database and Boolean operators were used (Table 1).

**TABLE 1:** Inclusion and exclusion criteria.

Topic	Inclusion criteria	Exclusion criteria
Population	Nursing staff, nursing teacher	Patient
Context	Intersectionality, discrimination, racism	No reference to intersectionality, discrimination, racism
Language	German, English	No German or English sources

The search string used was “intersectionality AND (“Nursing”[MeSH] OR “Education, Nursing”[MeSH]) AND (“Professional Development” OR Workplace) NOT (patient OR “patient care” OR “clinical outcomes”).”

The database search was conducted between 5 and 15 January 2025.

**FIGURE 1:**
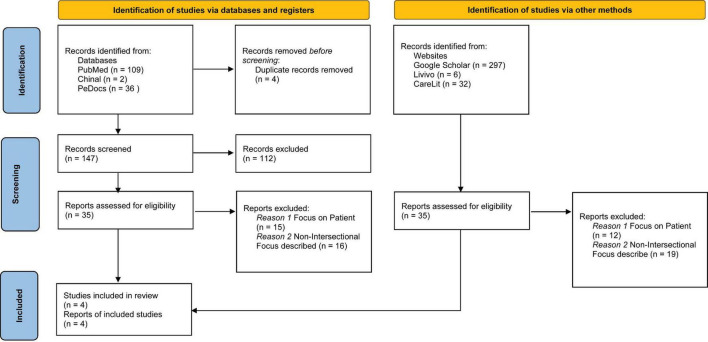
PRISMA flow chart of literature search and selection (20).

As part of the further search, a total of 70 studies were selected and evaluated for their suitability for the critical review. As part of an evaluation of the referenced studies, eight articles were identified as relevant (Figure 1) (six in English and two in German) and included in the results of the critical review.

The content was analyzed according to relevant conceptual and fundamental findings.

## Results

The analysis revealed a number of interlinked factors that shape intersectional inequalities in nursing education. Some important factors can be divided into influences stemming from international backgrounds and characteristics relevant to education, as shown in Figure 1 and Supplementary material (14).

### Intersectionality as a framework for nursing education

The findings highlight intersectionality as a key concept for enabling trainees and students to critically reflect on their role within the power structures of the healthcare system. Taking intersectional perspectives into account makes it possible to recognize and challenge discrimination based on gender, ethnicity, religion and other social categories. At the same time, insufficient integration of these perspectives carries the risk of reproducing existing inequalities in educational and institutional contexts (21).

It is evident, therefore, that nursing education is shaped by hegemonic structures that often reflect a predominantly Eurocentric and white middle-class perspective. These dynamics influence not only the content of education but also career trajectories, as institutional practices can implicitly steer students along racialized lines into different professional paths (22, 23).

Ethical aspects of nursing education are also closely linked to intersectional awareness. Without an explicit engagement with social inequalities, students may be inadequately prepared to navigate ethical dilemmas in diverse nursing contexts (24).

### Discrimination and language-related barriers in educational settings

Overall, it can be argued that discrimination within the healthcare system occurs at multiple levels and manifests itself across overlapping – intersectional – categories such as gender, international background, language and socio-economic status (25). These dynamics contribute to structural barriers that limit access to professional qualifications and further training opportunities for nurses with an international background (1).

Institutional hierarchies play a central role in the reproduction of these inequalities. Nurses with an international background are often placed in lower-status positions within the profession, which limits career progression and cements existing power structures. This placement is further shaped by dependencies related to residency status and employment, which can limit an individual’s ability to challenge discrimination or pursue alternative career paths (25).

Furthermore, the findings demonstrate how intersectional factors influence access to educational opportunities in a broader sense. Nurses with an international background frequently and migration story encounter obstacles in the recognition of prior qualifications, leading to delayed or restricted access to further training and professional development (14).

### Structural inequalities in access to qualifications and the gender care gap

Related factors of international biographie form a central dimension of intersectional inequalities in nursing education (25). These include the reasons for migration (e.g., labor migration or forced migration), country of origin, duration of stay in the new country, and prior educational experiences (14, 26).

These dimensions influence access to the education system, familiarity with institutional structures, and the recognition of prior qualifications. Differences in educational biographies and systemic alignment can create additional barriers, particularly when prior learning is not fully recognized or transferable (14).

Furthermore, the duration of stay and degree of social integration affect individuals’ ability to navigate educational and professional systems (21), while cultural and linguistic distance may further complicate access to learning opportunities.

**FIGURE 2:**
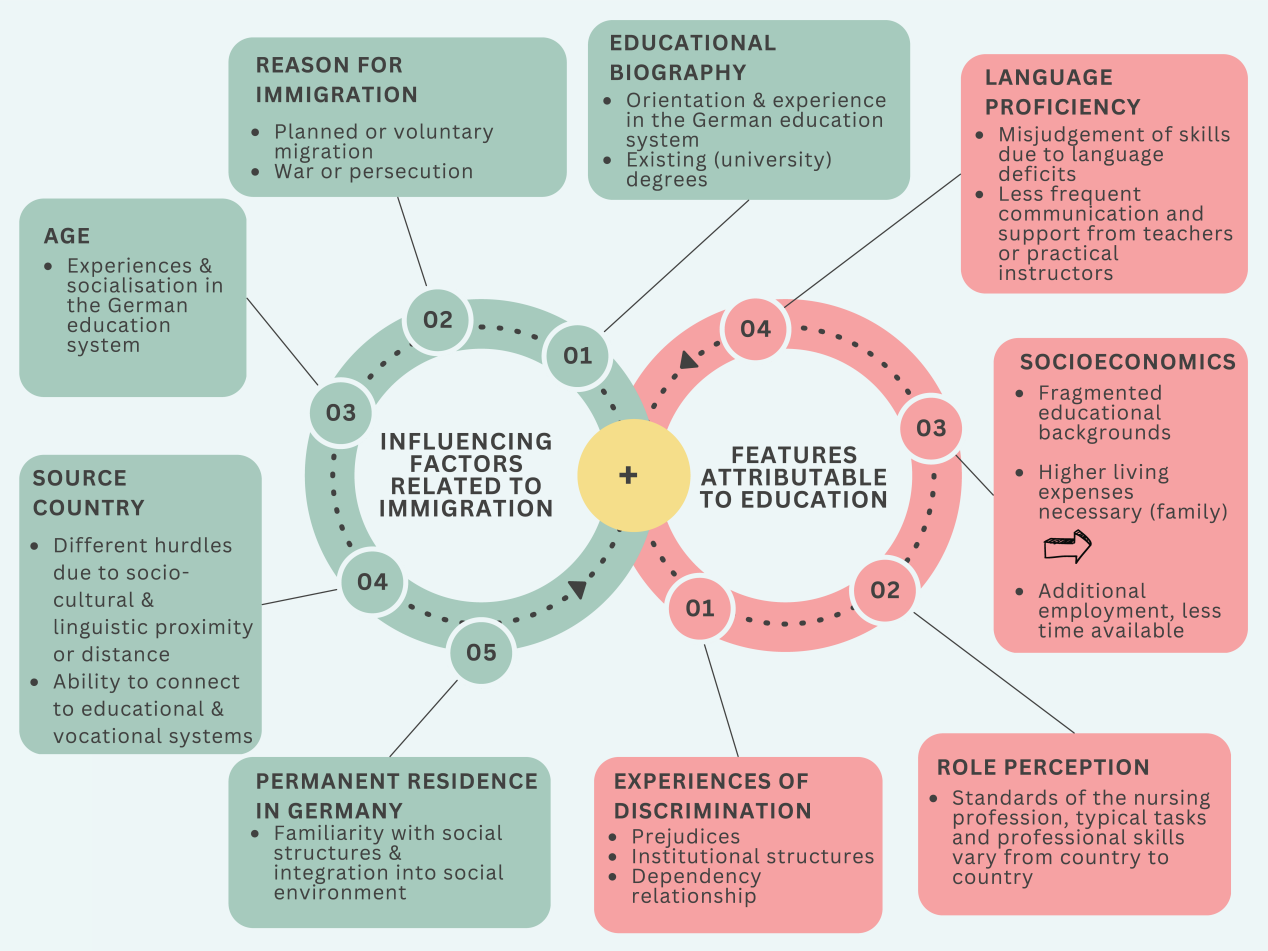
Factors influencing nurses with international backgrounds and migration (14) (own representation, Canva). The figure illustrates how immigration-related structural conditions intersect with education-related and institutional dynamics, thereby shaping trainees’ experiences and their access to learning opportunities. It is important to note that these factors do not operate independently of one another, but rather reinforce each other, leading to cumulative forms of disadvantage. This graphic was created using Canva. The illustrations and graphic elements it contains (e.g. icons, avatars) are not licensed under CC BY-SA 4.0. They are authorised for use solely within this publication and may not be reused or extracted separately. All other content in this publication is licensed under CC BY-SA 4.0, unless otherwise indicated.

Access to educational opportunities in the health care sector is also characterized by intersectional inequalities, which illustrate that immigrant health care workers are systematically disadvantaged (25). Women, as the main group of international health care workers (10), face multiple forms of discrimination resulting from gender-specific, racist, and socio-economic factors. Women in particular are disproportionately affected by the unequal distribution of unpaid care work (gender care gap), which places additional demands on their time and resources alongside professional training. For example, qualitative findings show that trainees often manage extensive private care responsibilities – such as childcare, coordinating medical appointments, and emotional support – (Figure 2 “Socioeconomics) while simultaneously coping with the demands of nursing education, leading to exhaustion, reduced learning capacity, and financial strain (27). These intersectional inequalities are further exacerbated by the hierarchical structure of the healthcare system, which means that nurses with a migration background often work in poorly paid positions and have limited access to further training and qualification measures (25).

### Digitalization and intersectional inequalities

Digitalization gives rise to further intersectional dynamics. Existing digital strategies are often insufficiently adapted to the needs of different user groups, which can exacerbate existing inequalities. Factors such as gender and age influence both the perception and use of digital technologies, which has implications for the development of digital competence and participation in digital transformation processes (19).

At the same time, limited involvement of nursing staff in the implementation of digital technologies can contribute to resistance and lower acceptance. This is particularly relevant when digital tools are perceived as an additional burden rather than as meaningful support for practice (19).

## Discussion

Rather than being primarily caused by individual deficits, unequal access to nursing education and professional development appears to be largely produced by structural and institutional conditions. This review’s findings suggest that language-related exclusion, delayed recognition of prior qualifications, unequal care responsibilities, and hierarchical power relations interact and reinforce each other (13, 14, 25). This suggests that intersectionality is an important framework for understanding how educational opportunities are shaped by the interaction of multiple dimensions of inequality, rather than merely an additional category within nursing education (3, 21).

While most studies identify comparable barriers, the emphasis varies depending on the national and disciplinary context. For example, studies from North America predominantly focus on race, institutional racism, and the Eurocentric orientation of nursing education (22, 23), whereas German-language studies place greater emphasis on language-related exclusion, recognition of qualifications, and the gender care gap (14, 18, 27). These differences suggest that intersectional inequalities are context-dependent, shaped by the organization of national healthcare and education systems (1). However, the available evidence is limited by the predominance of qualitative and cross-sectional research designs. Only a small number of studies examine the long-term consequences of intersectional inequalities for educational success, career progression, or workforce retention. Similarly, there is still limited evidence from low- and middle-income countries, despite the increasing internationalization of the nursing workforce (12, 25). Therefore, the transferability of existing findings to the German healthcare system must be approached with caution, particularly given that nursing education in Germany differs considerably from predominantly university-based systems in many other countries (1).

Nevertheless, the findings have important implications for nursing education and policy. Curricula should address cultural differences in relation to patients and the structural inequalities experienced by nursing professionals. This should include explicit engagement with racism, sexism, class-related disadvantage, language-related exclusion, and the unequal distribution of unpaid care work (24, 28). Furthermore, educators require support and training to recognize how institutional routines and teaching practices may unintentionally perpetuate exclusion (21).

The issue of language requirements illustrates this particularly clearly. Current legislation, such as the German Training and Examination Regulations for Nursing Professions (29), emphasizes German language proficiency as a prerequisite for professional practice. However, the literature shows that educational language requirements, such as understanding examination terminology, academic language, and profession-specific communication, are rarely addressed systematically (30). Consequently, language-related barriers are often perceived as personal shortcomings rather than structural challenges for which educational institutions are also responsible (30).

Findings on digitalization further demonstrate that digital technologies are not neutral tools. Existing digitalization strategies often fail to consider the needs of different user groups and may therefore perpetuate existing inequalities (19). In particular, gender, age, and educational background influence the perception of, and the ability to use, digital technologies. If nursing staff are not involved in developing and implementing digital tools, digitalization may reinforce exclusion rather than reduce it (11, 19).

## Limitations

It is important to acknowledge that this critical review does not claim to provide a comprehensive account of the topic. This limitation is partly due to the scope defined by the research question, as well as the selection of search operators and filters applied during the literature review process. Given the limited availability of German-language scholarship on the subject, the review increasingly drew on English-language sources. However, cross-national comparisons remain constrained, as professional nursing qualifications are predominantly obtained through university education in many countries. Further research is required – particularly within the context of the German healthcare and education systems – to develop targeted recommendations for action and viable implementation strategies.

## Conclusion

Overall, the integration of intersectional perspectives into qualification frameworks, both in the training of nursing students and in fostering professional-ethical reflection on power relations, emerges as a meaningful and necessary step. The absence of such integration contributes to the perpetuation of discriminatory institutional structures, with detrimental effects on both nurses and patients. Embedding intersectional awareness into qualification, onboarding, and integration processes for nursing professionals holds the potential to initiate structural change within the existing hierarchies of the healthcare system.

## Supplementary material

Supplementary Table 1 Summary table of the topics, methods, and results of the included studies.
