# Supplementary material for: Nursing education in conflict: How intersectionality impacts access to educational opportunities?
Source: Front Med (Lausanne). 2026 May 11;13:1804741. doi: 10.3389/fmed.2026.1804741 (PMC13199105; doi:10.3389/fmed.2026.1804741)
Supplement: Supplementary Table 1 — Summary table of the topics, methods, and results of the included studies. [file Table_1.docx]

| Study | topic | method | results relating to the research question |
| --- | --- | --- | --- |
| Ion, R.; DeSouza, R.; Kerin, T. (2018): Teaching ethics: Intersectionality, care failure and moral courage [14] | Necessity of incorporating ethical principles, intersectionality, and training  The working environment as a space for critical reflection | feature article | Intersectionality, as a departure from the monothematic interpretation of ethical principles, opens a new perspective that analyses power structures, inequalities, and social categories in relation to health problems and experiences of nursing care. |
| Younas, Ahtisham; Monari, Esther N.; Ali, Parveen (2024): Applying intersectionality to address inequalities in nursing education [12] | Possibility of analysing power structures by integrating intersectionality and reflecting on one's own prejudices and stereotypes in the qualification of carers, care teachers, trainees, and students. | Discussion paper | Reflection on power structures in nursing education.  Criticises the Eurocentric orientation of curricula, which contributes to discrimination against marginalized groups, and emphasis the importance of intersectional approaches to promoting equal opportunities. The integration of intersectional perspectives into nursing qualifications can help to identify and reduce socio-cultural inequalities. |
| Daase A, Fleiner M (2024) Linguistic challenges and needs from the perspective of learners in the context of generalist nursing training [16] | Addressing increasing linguistic heterogeneity is presented as a necessary topic in nursing education, partly because insufficient awareness among teachers can lead to discrimination. | Research project description | Linguistic heterogeneity can be considered an intersectional factor that can reveal discrimination.  Language-conscious approaches in education and training are an important prerequisite for equal opportunities, not least because they raise awareness among teachers. |
| Olden, Daria; Großmann, Daniel; Reuschenbach, Bernd (2024): Challenges faced by trainees with a migrant background in generalist nursing training [10] | Presentation of the challenges faced by trainees with a international background in generalised nursing training (sociocultural, financial, linguistic, experience of discrimination) in Germany | Book chapter | nursing students with a international background are structurally disadvantaged in nursing due to intersectional factors such as language, educational background and financial dependence. These challenges make it difficult for them to access educational opportunities and require targeted support measures, which have been insufficient to date |
| Neiterman E, Bourgeault IL (2015) The shield of professional status: Comparing internationally educated nurses' and international medical graduates' experiences of discrimination.[17] | Reflection on the role that gender and professional status play in the experiences of ethnic discrimination among internationally trained healthcare professionals, also differences between doctors and nurses. | 140 semi-structured qualitative interviews with internationally  trained nurses and doctors after their migration to the Canadian healthcare system | Nursing with a international background experiences systematic discrimination based on intersectional categories such as gender, origin and language. Hierarchical structures in the healthcare system in particular reinforce these inequalities by pushing international nursing staff into lower positions and making it more difficult for them to access education. |
| Garneau AB, Browne AJ, Varcoe C (2018) Drawing on antiracist approaches toward a critical antidiscriminatory pedagogy for nursing [15] | Integration of anti-discriminatory pedagogy into the qualification for carers for transformative learning and critical intersectional reflection | magazine article | The need for a critical anti-discriminatory pedagogical concept (CADP) in nursing education to recognise and combat systemic discrimination and intersectional inequalities in healthcare. Integrating intersectional perspectives into teaching promotes awareness of power structures and social inequalities and strengthens the reflective abilities of teachers and learners. |
| Wynn M, Garwood-Cross L, Vasilica C, Griffiths M, Heaslip V, Phillips N (2023) Digitizing nursing: A theoretical and holistic exploration to understand the adoption and use of digital technologies by nurses [18] | Barriers to developing digital skills in nursing (age, gender) | Discursive analysis using literature research | The integration of digital technologies in nursing is impacted by intersectional categories such as gender and age. Gender roles and low social pressure in older age have a negative impact on usage, while hierarchical structures and a lack of adaptation to user needs create barriers. |
| van Herk KA, Smith D, Andrew C (2011) Examining our privileges and oppressions: incorporating an intersectionality paradigm into nursing.[13] | Integration of the intersectionality paradigm into nursing care | Narrative analysis | The intersectionality paradigm is an important tool for analysing power structures in nursing. It highlights how hegemonic perspectives, particularly the “white, middle-class” norm, shape nursing research, practice, and education and perpetuate structural inequalities. |

*The datasets used and/or analysed during the current study are available from the corresponding author on reasonable request.*

Literature

1. Daase A, Fleiner M (2024) Sprachliche Herausforderungen und Bedürfnisse aus Sicht von Lernenden im Kontext der generalistischen Pflegeausbildung – Einblicke in das erste Ausbildungsjahr. Zeitschrift für Interkulturellen Fremdsprachenunterricht Volume 29 Issue 1 2024. https://doi.org/10.48694/ZIF.3901

2. Garneau AB, Browne AJ, Varcoe C (2018) Drawing on antiracist approaches toward a critical antidiscriminatory pedagogy for nursing. Nurs Inq 25(1). https://doi.org/10.1111/nin.12211

3. Ion R, DeSouza R, Kerin T (2018) Teaching ethics: Intersectionality, care failure and moral courage. Nurse Educ Today 62:98–100. https://doi.org/10.1016/j.nedt.2017.12.023

4. Neiterman E, Bourgeault IL (2015) The shield of professional status: Comparing internationally educated nurses' and international medical graduates' experiences of discrimination. Health (London) 19(6):615–634. https://doi.org/10.1177/1363459314567788

5. Olden D, Großmann D, Reuschenbach B (2024) Herausforderungen von Auszubildenden mit Migrationshintergrund in der generalistischen Pflegeausbildung. In: Reiber KE, Mohr J, Evans-Borchers M et al. (Hrsg) Fachkräftesicherung, Versorgungsqualität und Karrieren in der Pflege. Forschung zur beruflichen Bildung im Lebenslauf. wbv, Bielefeld, S 319–336

6. van Herk KA, Smith D, Andrew C (2011) Examining our privileges and oppressions: incorporating an intersectionality paradigm into nursing. Nurs Inq 18(1):29–39. https://doi.org/10.1111/j.1440-1800.2011.00539.x

7. Wynn M, Garwood-Cross L, Vasilica C, Griffiths M, Heaslip V, Phillips N (2023) Digitizing nursing: A theoretical and holistic exploration to understand the adoption and use of digital technologies by nurses. J Adv Nurs 79(10):3737–3747. https://doi.org/10.1111/jan.15810

8. Younas A, Monari EN, Ali P (2024) Applying intersectionality to address inequalities in nursing education. Nurse Educ Pract 77:103982. https://doi.org/10.1016/j.nepr.2024.103982
